# Template for *MSM* Submissions

**Published:** 2011

**Authors:** Ajai R. Singh

**Affiliations:** Editor, *MSM*, India mensanamonographs@yahoo.co.uk

This is a brief write-up for *MSM* authors meant to be a check-list to adhere to in their submissions.

While writing, do look into the following:

1. Add an **Abstract** at the beginning [100-150 words].
2. Add **Key Words** after Abstract [4-6 key words from the text], separated by semi-colons.
3. Add **Author Affiliations**, Correspondence address, and email id below Key Words.
4. Start article with **Introduction**. Break the write-up into suitable paragraphs, with headings and subheadings as required.
5. Add **Flowchart of the paper**, at the end of the write up but before Concluding Remarks, as a figure that includes salient features of the paper and its important findings, so readers can get a pictorial overview of the paper and the thought as it flows through the text. This helps both the reader understand the author and helps the author clarify his thoughts, both for the reader, and most importantly, for himself. For flowchart, see any *MSM* paper 2010 onwards.
6. Add **Concluding Remarks** [100-150 words in 1-2 paragraphs which conclude the arguments and put things in a nutshell for the reader].
7. Add **Take Home message** [3-4 lines] after concluding remarks. Take home should be the absolute gist of the paper and less than concluding remarks.
8. Add **Conflict of Interest [COI] statement**, if any, after Take Home message [If none, say so. COI statement is to acknowledge any funding/sponsorship etc by any agency which could be construed to have influenced the write-up - this is essential for readers to know, especially as some authors, especially in scientific papers, are funded by agencies which may have a direct/indirect interest in influencing results/write-ups].
9. Add **Declaration** that it is your original unpublished work, not submitted for publication elsewhere.
10. Add **acknowledgements**, if any, after Declaration
11. Points 1-10 are mandatory requirements for any paper, including my own.
12. **Why Concluding remarks and Take Home?**
  The reason why we insist on Concluding remarks and Take Home is to present the final summary and absolute gist of the paper to
  - Encourage the reader to give the paper a second read.
  - Re-understand the paper in a clearer manner after reading it the first time: often so many ideas are presented in the text of the paper that the reader may be drowned in attempting to make sense; Concluding remarks and Take home message help a reader get a clearer grasp.
  - Start the first reading of the paper with some grounding in what the paper is trying to essentially say.
  - Help clarify the author’s thoughts to himself.
    All these methods make the author intelligible, the paper readable, and the thoughts digestible.
13. Add **Questions that the Paper Raises** (4-7 questions that readers/researchers can deliberate over in the light of your write-up and to advance future research in the field; they are research type, not text book or CME type, questions), after Acknowledgement/ Declaration.
14. Add 100-150 words **About the author** with a recent high-resolution jpg photo to go along with your write-up, after Questions that the Paper Raises
15. Add **References** in Modified Harvard style [see References below, point 22, or an article at *MSM* website www.msmonographs.org for details] after **About the author**.
16. **Full length article**: A paper may ordinarily not be more than 4500 words, complete with an abstract (150 words maximum), introduction and concluding remarks (150 words maximum). Each Article may contain not more than 30 references, 50% of which are 2000 AD or after. [This is so that recent work does not go unnoticed.]
17. **Editorial**: An editorial may ordinarily not be more than 2500 words with max 30 references [50% of which are 2000 or later].
18. **Feature and Communications**: *Musings* max 1500 words. Reflections, The Looking Mirror max 2500 words, with 20 references [50% 2000 or later]. Letters to editor word limit is 1000 words. *MSM* Book review word limit is 2000 words.
19. All submissions in **Microsoft WORD**, Times New Roman 12pt font size.
20. **Review**: Submissions are subject to editorial and peer review.
21. **Deadlines**: Authors are advised to stick to deadlines of submissions strictly. As also carry out editorial corrections and peer review modifications as per time schedule given by the editorial office as follows: [The deadline for this submission is: …]
22. **References**
  Authors must check authenticity of references and other facts quoted before submission. They must specially check that all references in text are included in reference list, and vice versa. No reference not in text should occur in the reference list. Do not prepare a separate Bibliography or Internet Citation list. They should be incorporated in the reference list itself, and only if quoted in the text.
  In the text, references should occur with only surnames, and in the following form e.g. for one name (Fulford, 2011); for two names (Singh and Singh, 2011); for more than 2 names (Drubach *et al*., 2011).
  Reference style is Harvard [modified as per *MSM*].

All References should be bunched together in alphabetical order at the end of the submission. They should follow the following pattern:

- ***For References of Same Author in One Year***:
  For two or more references from the same author/authors in the same year, (a) and (b) maybe used after the year of publication. For example:
  **Woodruff T., (2004a)**, Letters: The medical profession and the pharmaceutical industry: when will we open our eyes? *eMJA*, **181**(8), p458-459.
  **Woodruff T.G., (2004b)**, Pharmaceutical marketing, the PBS, and patient care, *New Doctor*, **81**, p21-22.
- ***For Editorials***:
  **Angell M., (2000)**, Is Academic Medicine for Sale? (Editorial), *N Engl J Med*, **342**(20), p1516-1518.
- ***For Papers***:
  iii.a. For a single author
  **Schafer A., (2004)**, Biomedical conflicts of interest: a defence of the sequestration thesis-learning from the cases of Nancy Olivieri and David Healy, *J Med Ethics*, **30**, p8-24.
  iii.b. For more than one author [all names preferred; *et al* may be used only after naming 6 authors]
  **Phillips B., Nylander K., Harnaha J., Machen J., Lakomy R., Styche A., Gillis K., Brown L., Lafreniere D., Gallo M., Knox J., Hogeland K., Trucco M., Giannoukakis N., (2008)**, A microsphere-based vaccine prevents and reverses new-onset autoimmune diabetes, *Diabetes*, **57** (6), p1544-1555.
  **Or**
  **Phillips B., Nylander K., Harnaha J., Machen J., Lakomy R., Styche A.,** *et al*., **(2008)**, A microsphere-based vaccine prevents and reverses new-onset autoimmune diabetes, *Diabetes*, **57** (6), p1544-1555.
- For Books and Monographs:
  ***iv.a. Reference of a book***
  i) **Baars B., (1988)**, *A Cognitive Theory of Consciousness*. Cambridge: Cambridge University Press.
  ***iv.b. Reference of a monograph series***
  ii) **Singh A.R., Singh S.A., (2005)**, Medical Practice, Psychiatry and the Pharmaceutical Industry: And Ever the Trio shall Meet-I: The Connection between Academia and Industry, *Mens Sana Monographs*, **II:** (6), **III:** (1), March-June, p5-35.
  ***iv.c. Where a book chapter is referenced***
  **Dulany D.E., (2009)**, Psychology and the study of consciousness. In T. Bayne, A. Cleeremans, P. Wilken (Eds.), *The Oxford Companion to Consciousnes*, (p540-544). Oxford, UK: Oxford University Press.
  ***iv.d. Where an encyclopedia entry is referenced***
  **Shaffer J., (1972)**, Mind-Body Problem. In: Paul Edwards [Ed], *The Encyclopedia of Philosophy*, Vol 5 & 6, p343. New York: Macmillan.
  ***iv.e. Where a book in translated***
  **Heidegger M., (1927/1962)**, *Being and Time* (Sein und Zeit). Translated by J. Macquarrie and E. Robinson. New York: Harper and Row.
  ***iv.f. Where an older book is republished***
  Heidegger M., (1927/1962), Being and Time (Sein und Zeit). Translated by J. Macquarrie and E. Robinson. New York: Harper and Row.
  ***iv.g. Where a book has been edited***
  **Flew A., (Ed.) (1983)**, *A Dictionary of Philosophy*. London: Pan Books in association with The Macmillan Press.
  ***iv.h Where a book has been edited by someone other than the author***
  **Hume D., (1739/1888)**, A Treatise of Human Nature. (Ed.) L Selby-Bigge. Oxford: Oxford University Press.
  ***iv.i. Where a book has been published by 2 publishers***
  **Flew A., (1983, ed)**, *A Dictionary of Philosophy*. London: Pan Books in association with The Macmillan Press.
  ***iv.j. Where a book is published by one but reprinted by another***
  **James W., (1890/1999)**, *The Principles of Psychology*. Ch 9. (2 vols.). New York: Henry Holt (Reprinted Bristol: Thoemmes Press, 1999).
- For Web References:
  **National Institute of Mental Health, (2011)**, Schizophrenia. Available at http://www.nimh.nih.gov/health/topics/schizophrenia/index.shtml [Accessed 20 Dec 2010.].
- For News Paper/Magazine Articles:
  **Harris G., (2004)**, As doctor writes prescription, drug company writes a check, *New York Timesc*, June 27, A1.
- Personal communications
  Personal communication may not ordinarily be quoted as a reference. If at all done, it should be with the written permission of the communicator.

21. **Copyright**

Copyright of material published rests with the *Mens Sana Monographs*. However, authors are permitted to disseminate their published work for non-commercial purposes freely by post or electronic means, and to put it up on their personal/institutional web sites for the information and knowledge of their viewers and fellow researchers, with due acknowledgement of the original source of publication (*MSM*). Authors should contact and obtain permission from *MSM* if they intend publication of their work in any other form later. Such requests are ordinarily granted on due acknowledgement of publication in *MSM*.

22. **Typical Paper, Editorial, and other submissions**

For a typical paper, see
  http://www.msmonographs.org/article.asp?issn=0973-1229;year=2009;volume=7;issue=1;spage=10;epage=19;aulast=Chadwick
  And/or
http://www.msmonographs.org/article.asp?issn=0973-1229;year=2010;volume=8;issue=1;spage=30;epage=51;aulast=Varma
For a typical editorial, see
  http://www.msmonographs.org/article.asp?issn=0973-1229;year=2010;volume=8;issue=1;spage=6;epage=16;aulast=Schwartz
For a typical Book review, see
  http://www.msmonographs.org/article.asp?issn=0973-1229;year=2010;volume=8;issue=1;spage=146;epage=150;aulast=Andrade
For typical *MSM* Poem, see
  http://www.msmonographs.org/article.asp?issn=0973-1229;year=2008;volume=6;issue=1;spage=277;epage=278;aulast=Brenner
For a typical Journalology article, see
  http://www.msmonographs.org/article.asp?issn=0973-1229;year=2008;volume=6;issue=1;spage=226;epage=236;aulast=Hoey
For a typical article in the Looking Glass, see
  http://www.msmonographs.org/article.asp?issn=0973-1229;year=2008;volume=6;issue=1;spage=41;epage=47;aulast=Gilman
For a typical article in Reflections, see
  http://www.msmonographs.org/article.asp?issn=0973-1229;year=2008;volume=6;issue=1;spage=135;epage=145;aulast=Narlikar
For a typical article in Musings, see
  http://www.msmonographs.org/article.asp?issn=0973-1229;year=2008;volume=6;issue=1;spage=131;epage=134;aulast=Arnold
For typical obituary, see
  http://www.msmonographs.org/article.asp?issn=0973-1229;year=2008;volume=6;issue=1;spage=281;epage=284;aulast=Raveh

Best wishes.

